# Sanggenon C inhibits cell proliferation and induces apoptosis by regulating the MIB1/DAPK1 axis in glioblastoma

**DOI:** 10.1002/mco2.281

**Published:** 2023-06-19

**Authors:** Hongbo Chang, Jianbing Hou, Yaqian Shao, Minghao Xu, Xuelian Weng, Yi Du, Junbo Shi, Li Zhang, Hongjuan Cui

**Affiliations:** ^1^ State Key Laboratory of Resource Insects Medical Research Institute Southwest University Chongqing China; ^2^ Department of Radiology and Nuclear Medicine The First Hospital of HeBei Medical University Hebei China; ^3^ Jinfeng Laboratory Chongqing China

**Keywords:** chemosensitivity, DAPK1, glioblastoma, MIB1, sanggenon C

## Abstract

Sanggenon C (SC), a herbal flavonoid extracted from Cortex Mori, has been mentioned to possess more than one treasured organic properties. However, the molecular mechanism of its anti‐tumor impact in glioblastoma (GBM) remains unclear. In this study, we reported that SC displayed a GBM‐suppressing impact in vitro and in vivo with no apparent organ toxicity. SC dramatically suppressed cell proliferation‐induced cell apoptosis in GBM cells. Mechanistically, we unveiled that SC modulated the protein expression of death associated protain kinase 1 (DAPK1) by controlling the ubiquitination and degradation of DAPK1. Quantitative proteomic and Western blot analyses showed that SC improved DAPK1 protein degradation via decreasing the expression of E3 ubiquitin ligase Mindbomb 1 (MIB1). More importantly, the effects of SC on cell proliferation and apoptosis of GBM cells have been in part reversed through DAPK1 downregulation or MIB1 overexpression, respectively. These results indicated that SC might suppress cell proliferation and induce cell apoptosis by decreasing MIB1‐mediated DAPK1 degradation. Furthermore, we found that SC acted synergistically with temozolomide (TMZ), an anti‐cancer drug used in GBM, resulting in elevated chemotherapeutic sensitivity of GBM to TMZ. Collectively, our data suggest that SC might be a promising anti‐cancer agent for GBM therapy.

## Graphical abstract

SC displayed a strong GBM‐suppressing effect in vitro and in vivo without obvious organ toxicity. SC can inhibit cell proliferation and induce cell apoptosis by decreasing MIB1‐mediated death associated protain kinase 1 (DAPK1) degradation, as well as enhance the chemosensitivity of TMZ to GBM, suggesting that SC might be a promising anti‐cancer agent for cancer therapy.

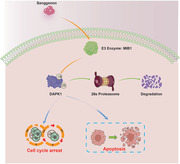

## 1 INTRODUCTION

Glioblastoma (GBM), the highest grade in the World Health Organization classification of brain gliomas, is the most common and fatal brain tumor with a dismal prognosis and poor quality of life.^1^ With the rise of modern biotechnology, such as genomics and proteomics, new therapeutic strategies based on the genetic and epigenetic profiling of GBM, brain microenvironment, and immune system interactions were discovered and put into clinical trials, including immune checkpoint blockade, chimeric antigen receptor T‐cell immunotherapy (CAR‐T) therapy, and oncolytic virotherapy.^2^, ^3^, ^4^, ^5^ However, GBM remains a malignancy with a median overall survival of 14.6–20.5 months due to the invasive and aggressive nature.^6^, ^7^ Given such a high burden of neurological morbidity, numerous researches are urgently needed to explore more effective therapeutic strategies, such as immunotherapy, drug delivery systems, and novel drugs.

*Morus alba* L. is often recognized as “mulberry” or “sang shu,” originated from China, and is extensively cultivated and naturalized in many nations as an indispensable herbal useful resource.^8^ It has been broadly used as a well‐known Chinese natural medication and possesses quite a few attainable pharmacological fitness advantages consisting of antioxidant, anti‐cholesterol, anti‐atherosclerosis, anti‐obesity, antihyperglycemic, immunomodulating, hypolipidemic, neuroprotective, and hepatoprotective effects, which would possibly be related to the existence of a number of bioactive compounds, such as flavonoids, anthocyanins, phenolic acids, flavonols, and unstable fragrant compounds.^9^, ^10^

Flavonoids, a major, well‐known plant secondary metabolite, have attracted more attention due to their valuable biological properties. One of the naturally occurring flavonoids derived from the Cortex Mori, Sanggenon C (SC), has been shown to have a number of beneficial biological and pharmacological qualities, including anti‐inflammatory, antioxidant, and anti‐cancer actions.^11^ In cardiac cells, SC displayed the cytoprotective effects against hypoxia stress via enhancing AMPKα activation and inhibiting the phosphorylation of mTOR and FOXO3a.^12^ Besides, SC possessed the neuroprotective effects against cerebral I/R injury by reducing the oxidative and inflammatory stress by targeting the RhoA‐ROCK signaling.^11^ Similarly, treatment with SC remarkably protected mesenchymal stem cells against oxidative stress by regulating multiple pathways.^13^ Hence, SC has been found to have clear anti‐oxidative and anti‐inflammatory effects in a number of illnesses.^14^ Interest in SC's anti‐cancer properties has grown recently as a result of in vitro and animal research showing that it slows tumor growth, including cell cycle and cell death. In human K562 cancer cells, SC displayed strong inhibitory effects by inducing cell cycle arrest and cell death via inhibiting tumor cellular proteasomal activity.^15^ In addition, another study documented that SC could dose‐dependently induce cell death by activating caspase3 and caspase9 signaling.^16^ However, the anti‐cancer effects of SC on biological role and underlying mechanism of tumors, especially GBM, are still unclear.

In this study, the anti‐cancer effect of SC on GBM was investigated. Our work indicated that SC suppressed cell proliferation and induced cell apoptosis by reducing Mindbomb 1 (MIB1)‐mediated death associated protain kinase 1 (DAPK1) protein degradation. Besides, SC remarkably enhanced the chemosensitivity of TMZ to GBM, suggesting that SC might be a promising anti‐cancer agent for cancer therapy.

## 2 RESULTS

### 2.1 SC suppresses GBM without obvious organ toxicity

**FIGURE 1:**
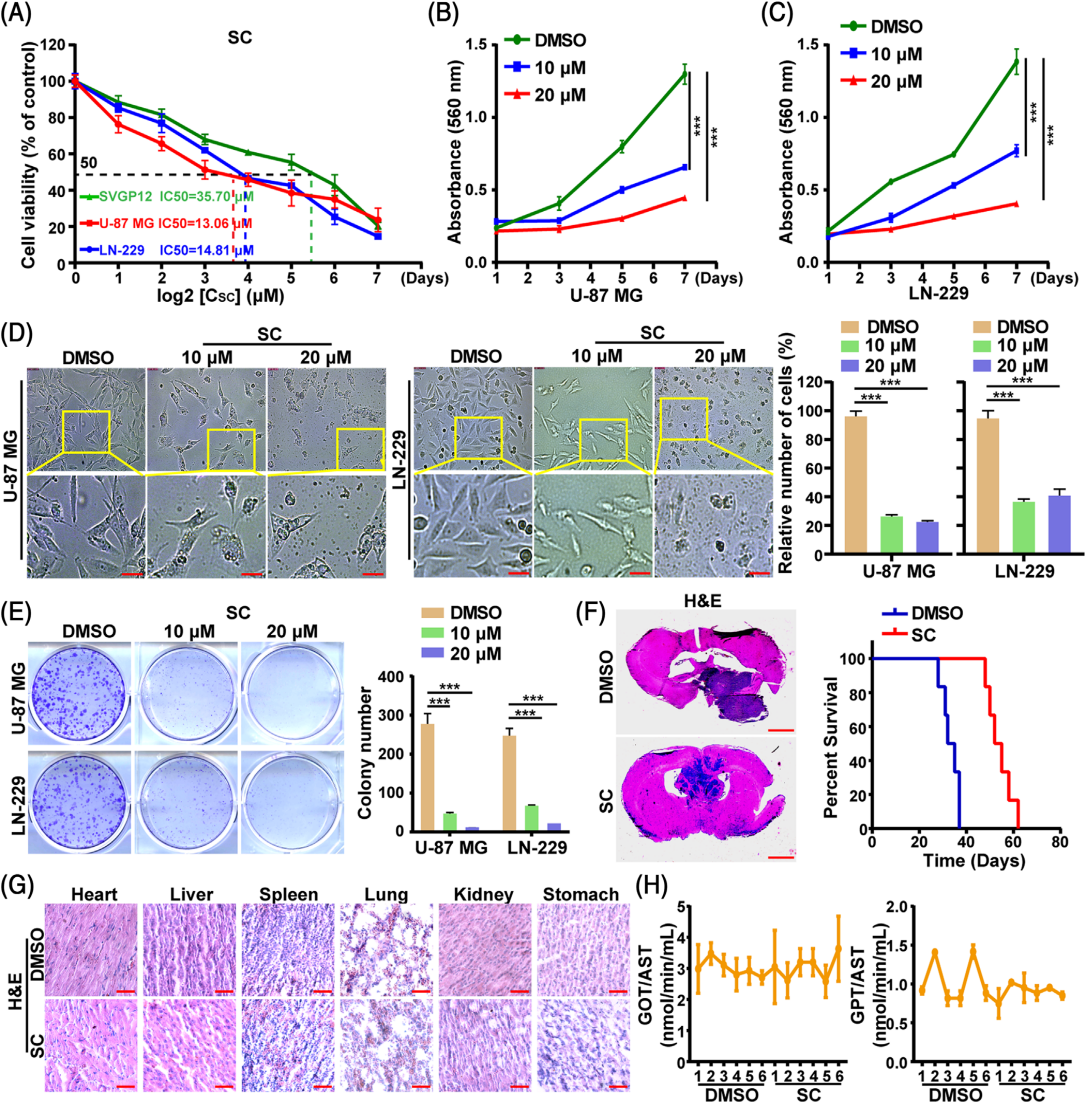
SC suppresses GBM without obvious organ toxicity. (A) GBM cells (U‐87 MG and LN‐229) and normal glial SVGP12 cells were treated with a series of indicated concentrations of SC for 2 days. MTT assays were used to detect cell viability. The IC50 values of SC in the tested cells were marked. DMSO was used as control. (B and C) Viability of U‐87 MG and LN‐229 cells after treatment with 10 and 20 μM SC. DMSO was used as control. (D) Morphology of U‐87 MG and LN‐229 cells after incubation with indicated concentrations of SC or DMSO for 48 h. Scale bars = 10 μm. DMSO was used as control. (E) The effects of indicated concentrations of SC or DMSO on the in vitro colony formation of U‐87 MG and LN‐229 cells. (F) Orthotopic implantation was performed after injection of U‐87 MG cells. Representative images of the hematoxylin and eosin (H&E). DMSO was used as control. H&E staining are presented. Scale bars = 2 mm. (G) H&E staining of the heart, liver, spleen, lung, kidney, and stomach in mice treated with SC or DMSO. Scale bars = 100 μm. (H) Serum levels of glutamic oxaloacetic transaminase/aspartate amino transferase (GOT/AST) and glutamic pyruvic transaminase/aspartate amino transferase (GPT/AST) in mice treated with SC or DMSO. DMSO was used as control. The data were expressed as mean ± standard deviation (SD). Student's *t*‐test was performed to analyzed significance. ^*^ *p* < 0.05, ^**^ *p* < 0.01, ^***^ *p* < 0.001.

Figure S1 displays the chemical formula for SC's structural makeup. To ascertain SC's impact on GBM cells, we treated U‐87 MG, LN‐229 and normal glial SVGP12 cells with a series of different concentrations of SC for 48 h. The results demonstrated that even relatively low concentrations of SC remarkably suppressed cell growth. Moreover, the median lethal concentration on SVGP12 was clearly higher than that on U‐87 MG and LN‐229 cells (Figure 1A). We gave the appropriate concentrations of SC (10 and 20 μM) to U‐87 MG and LN‐229, as well as dimethyl sulfoxide (DMSO) as a control, for 48 h. Using methyl thiazolyl tetrazolium (MTT) assays, it was demonstrated that SC had a dose‐dependent ability to inhibit the GBM cell lines U‐87 MG and LN‐229 (Figure 1B,C). Moreover, microscopic analysis showed that as SC concentration gradually increased, the number of U‐87 MG and LN‐229 living cells significantly decreased (Figure 1D). Next, the outcomes of an in vitro colony formation assay revealed that the SC group's capacity for colony formation was less than that of the control group (Figure 1E). Furthermore, the orthotopic implantation experiments in vivo indicated that the tumors in the SC group were significantly smaller as compared to those in the control group. SC therapy greatly reduced the ability of GBM cells to form tumors (Figure 1F,H). Most significantly, as seen in Figure 1G, mouse organs did not exhibit noticeable pathological changes; at the same time, kidney function and liver function were not significantly affected. These results suggest that SC had little toxicity to mouse visceral organs.

### 2.2 SC inhibits cell proliferation by inducing cell cycle arrest

**FIGURE 2:**
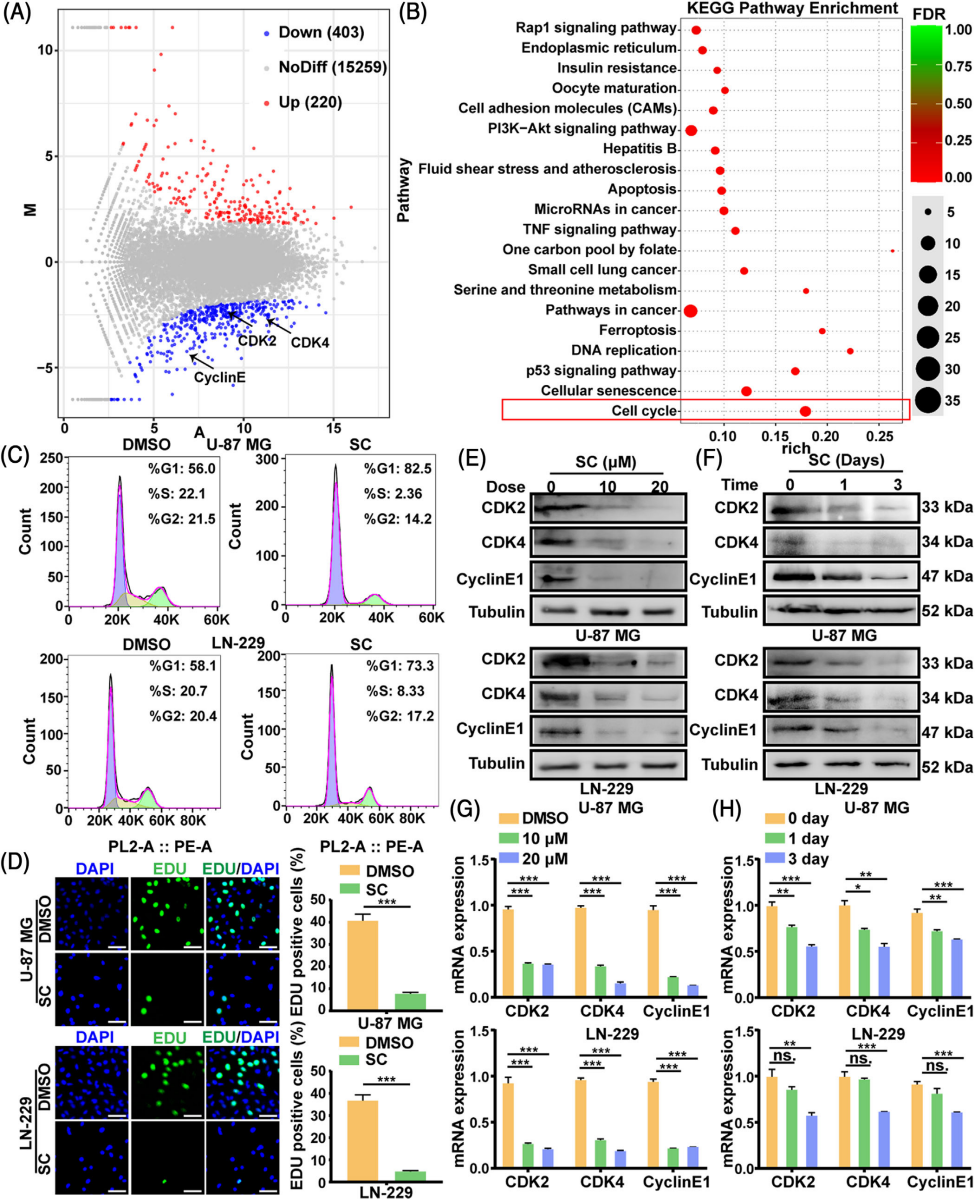
Sanggenon C (SC) inhibits cell proliferation by inducing cell cycle arrest. (A) Transcriptome analyses (RNA‐seq) in LN‐229 cells after treatment with SC (10 μM). The volcano plot to visualize the number of up‐ and downregulated genes is presented. (B) KEGG analysis of genes downregulated (determined via RNA‐seq data) in LN‐229 cells after treatment with SC. The top 20 KEGG pathway process terms based on fold enrichment are shown. DMSO was used as control. (C) The cell cycle of U‐87 MG and LN‐229 cells were detected via flow cytometry after treatment with SC (10 μM) or DMSO for 2 days. DMSO was used as control. (D) EDU‐positive U‐87 MG and LN‐229 cells after treatment with SC (10 μM) or DMSO for 2 days. Scale bars = 100 μm. DMSO was used as control. (E) Western blot assays were performed to detect the protein expression level of CDK2, CDK4, and CyclinE1 in U‐87 MG and LN‐229 cells treated with indicated concentration of SC for 2 days. DMSO was used as control. (F) Western blot assays were performed to detect the protein expression level of CDK2, CDK4, and CyclinE1 in U‐87 MG and LN‐229 cells treated with indicated time of SC (10 μM) for 2 days. DMSO was used as control. (G) RT‐PCR assays were performed to detect the mRNA expression level of CDK2, CDK4, and CyclinE1 in U‐87 MG and LN‐229 cells treated with indicated concentration of SC for 2 days. DMSO was used as control. (H) RT‐PCR assays were performed to detect the mRNA expression level of CDK2, CDK4, and CyclinE1 in U‐87 MG and LN‐229 cells treated with indicated time of SC (10 μM) for 2 days. DMSO was used as control. The data were expressed as mean ± standard deviation (SD). Student's *t*‐test was performed to analyzed significance. ^*^ *p* < 0.05, ^**^ *p* < 0.01, ^***^ *p* < 0.001.

According to the enrichment analysis of transcriptomics, it was found that the gene enrichment related to cell cycle was obvious after SC treatment (Figure 2A,B and Table S3). As shown in Figure 2C, cell cycle of U‐87 MG and LN‐229 cells was arrested at the G0/G1 phase in the SC group compared with the control. The 5‐ethynyl‐2‐deoxyuridine (EDU) assay results, which are depicted in Figure 2D, revealed that the SC group's cell proliferation was less than that of the control group. Subsequently, we identified the protein and mRNA expression of genes linked to the cell cycle. Western blot and ubiquitination experiments showed that p27 and p21 were upregulated in a dose‐dependent manner, and SC treatment significantly reduced the ubiquitination of p21 (Figure S2). Western blot and reverse transcription‐polymerase chain reaction (RT‐PCR) analyses showed that CDK2, CDK4, and CyclinE1 were downregulated in an SC dose‐ and time‐dependent manner (Figure 2E–H). Taken together, these results indicated that SC suppresses cell proliferation of GBM cells by inducing cell cycle arrest.

### 2.3 SC induces apoptosis in GBM cells

**FIGURE 3:**
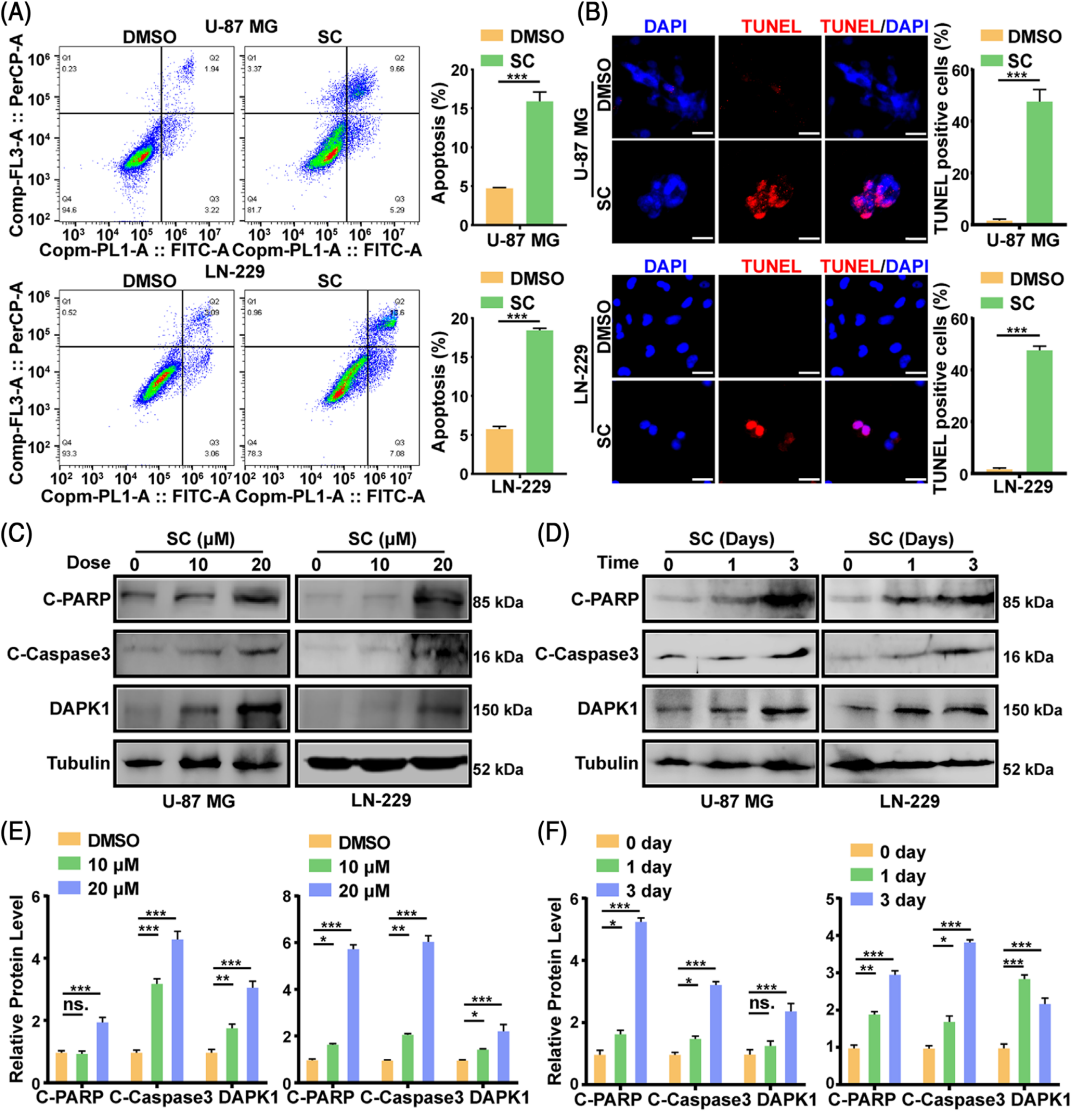
Sanggenon C (SC) induces apoptosis in glioblastoma (GBM) cells. (A) The apoptosis of U‐87 MG and LN‐229 cells treated with SC (10 μM) or DMSO for 2 days were detected by flow cytometry. DMSO was used as control. (B) Representative image of TUNEL staining of U‐87 MG and LN‐229 cells after treatment with SC (10 μM) or DMSO for 2 days. Scale bars = 50 μm. DMSO was used as control. (C and D) Western blot assays were performed to evaluate the apoptosis‐related proteins including cleaved poly ADP‐ribose polymerase (C‐PARP), cleaved caspase 3 (C‐Caspase3), and DAPK1 in the U‐87 MG and LN‐229 cells after treatment with indicated concentrations and indicated times of SC for 2 days. DMSO was used as control. (E and F) Protein levels are estimated based on the grayscale value of protein bands and normalized with the grayscale value of Tubulin bands. The data were expressed as mean ± standard deviation (SD). Student's *t*‐test was performed to analyzed significance. ^*^p < 0.05, ^**^ *p* < 0.01, ^***^ *p* < 0.001.

Apoptosis is a crucial factor for inhibiting cell proliferation. As shown in Figure 2B, the gene enrichment related to apoptosis was obvious after SC treatment. We discovered via flow cytometry that SC might significantly cause GBM cells to undergo apoptosis (Figure 3A). The terminal‐deoxynucleoitidyl transferase mediated nick end labeling (TUNEL) staining experiment consistently showed that SC did cause apparent apoptosis (Figure 3B). We discovered apoptosis‐related proteins, such as cleaved poly ADP‐ribose polymerase (C‐PARP), cleaved caspase 3 (C‐Caspase3), and death‐associated protein kinase 1, to further support our findings (DAPK1). The Western blot analysis revealed that C‐PARP, C‐Caspase3, and DAPK1 were raised in a manner dependent on SC concentration and time (Figure 3C–F). To explore whether SC treatment could induce autophagy in GBM cells, we performed the LC3B staining and found that SC treatment did not increase the LC3B staining in U‐87 MG and LN‐229 cells (Figure S3).

### 2.4 SC mediates cell proliferation and apoptosis of GBM cells by targeting DAPK1

**FIGURE 4:**
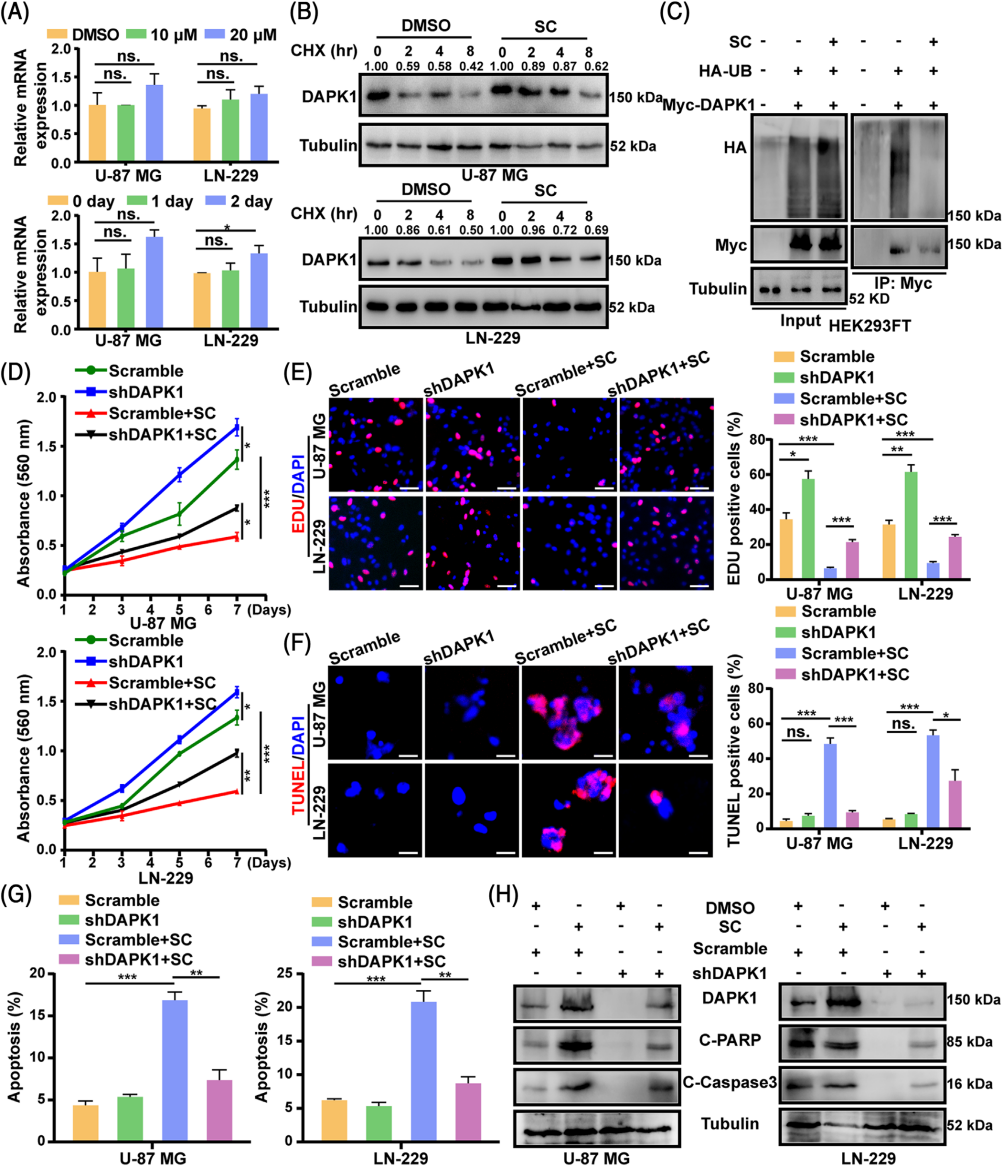
Sanggenon C (SC) mediates cell proliferation and apoptosis of glioblastoma (GBM) cells by targeting DAPK1. (A) RT‐PCR assays were performed to evaluate the mRNA expression level of DAPK1 in the U‐87 MG and LN‐229 cells after treatment with indicated concentrations and indicated times of SC for 2 days. DMSO was used as control. (B) U‐87 MG and LN‐229 cells were treated with SC (10 μM) or DMSO and were then treated with cycloheximide (CHX; 100 μg/mL) for the indicated times, and then were harvested and detect the DAPK1 turnover rate through Western blot analysis. The grayscale values were marked on the WB band, and 0 h was taken as the base for each group. DMSO was used as control. (C) The indicated plasmids were transfected into HEK293FT cells, and then treated with SC (10 μM) or DMSO. MG132 was added to the cells before harvested. The ubiquitinated DAPK1 proteins were pulled down with anti‐Myc antibody and immunoblotted with ant‐HA antibody. DMSO was used as control. (D) MTT assays were performed to determine cell proliferation of shDAPK1‐downregulation U‐87 MG and LN‐229 cells treated with SC (10 μM) or DMSO for indicated times. DMSO was used as control. (E) EDU‐positive cells in shDAPK1‐downregulation U‐87 MG and LN‐229 cells after treatment with SC (10 μM) or DMSO. Scale bars = 100 μm. DMSO was used as control. (F) TUNEL‐positive cells in shDAPK1‐downregulation U‐87 MG and LN‐229 cells after treatment with SC (10 μM) or DMSO. Scale bars = 50 μm. DMSO was used as control. (G) shDAPK1‐downregulation U‐87 MG and LN‐229 cells were treated with SC (10 μM) or DMSO for 2 days and apoptosis was determined by flow cytometry. The statistical analysis is presented in histograms. DMSO was used as control. (H) Western blot assays were performed to examine the protein expression level of DAPK1, cleaved poly ADP‐ribose polymerase (C‐PARP), and cleaved caspase 3 (C‐Caspase3) in shDAPK1‐downregulation U‐87 MG and LN‐229 cells after treatment with SC (10 μM) or DMSO for 2 days. DMSO was used as control. The data were expressed as mean ± standard deviation (SD). Student's *t*‐test was performed to analyzed significance. ^*^ *p* < 0.05, ^**^ *p* < 0.01, ^***^ *p* < 0.001.

We used RT‐PCR assays to further study the mechanism by which SC regulates the expression of DAPK1, and we discovered that it did not affect the level of DAPK1 mRNA expression (Figure 4A). As a result, we hypothesized that SC might control DAPK1's protein stability. SC could stabilize the DAPK1 protein by lowering its turnover rate when cycloheximide (CHX), an inhibitor of de novo protein synthesis, is present (Figures 4B and S4). We looked at DAPK1's level of ubiquitination to confirm the stabilizing capacity of SC on DAPK1. DAPK1 ubiquitination was significantly decreased by SC treatment (Figure 4C). Therefore, we presumed DAPK1 to be a critical factor in SC‐mediated cell proliferation and apoptosis. Next, we constructed stably expressed knockdown DAPK1 cell lines using lentivirus infection (Figure S5A). The MTT and EDU experiments proved that DAPK1 downregulation significantly increased cell proliferation of U‐87 MG and LN‐229 cells treated with SC (Figure 4D,E). TUNEL staining assays and flow cytometry experiments illustrated that silencing of DAPK1 decreased cell apoptosis induced by SC (Figures 4F,G and S5B). In addition, colony formation assays revealed that DAPK1 downregulation significantly increased the colony formation ability of SC‐treated U‐87 MG and LN‐229 cells compared with control cells (Figure S5C). Subsequently, Western blot analysis further indicated that the protein levels of DAPK1, C‐PARP, and C‐Caspase3 were decreased by silencing of DAPK1 in the SC‐treated GBM cells (Figures 4H and S5D).

### 2.5 SC increases the protein stability of DAPK1 through MIB1

**FIGURE 5:**
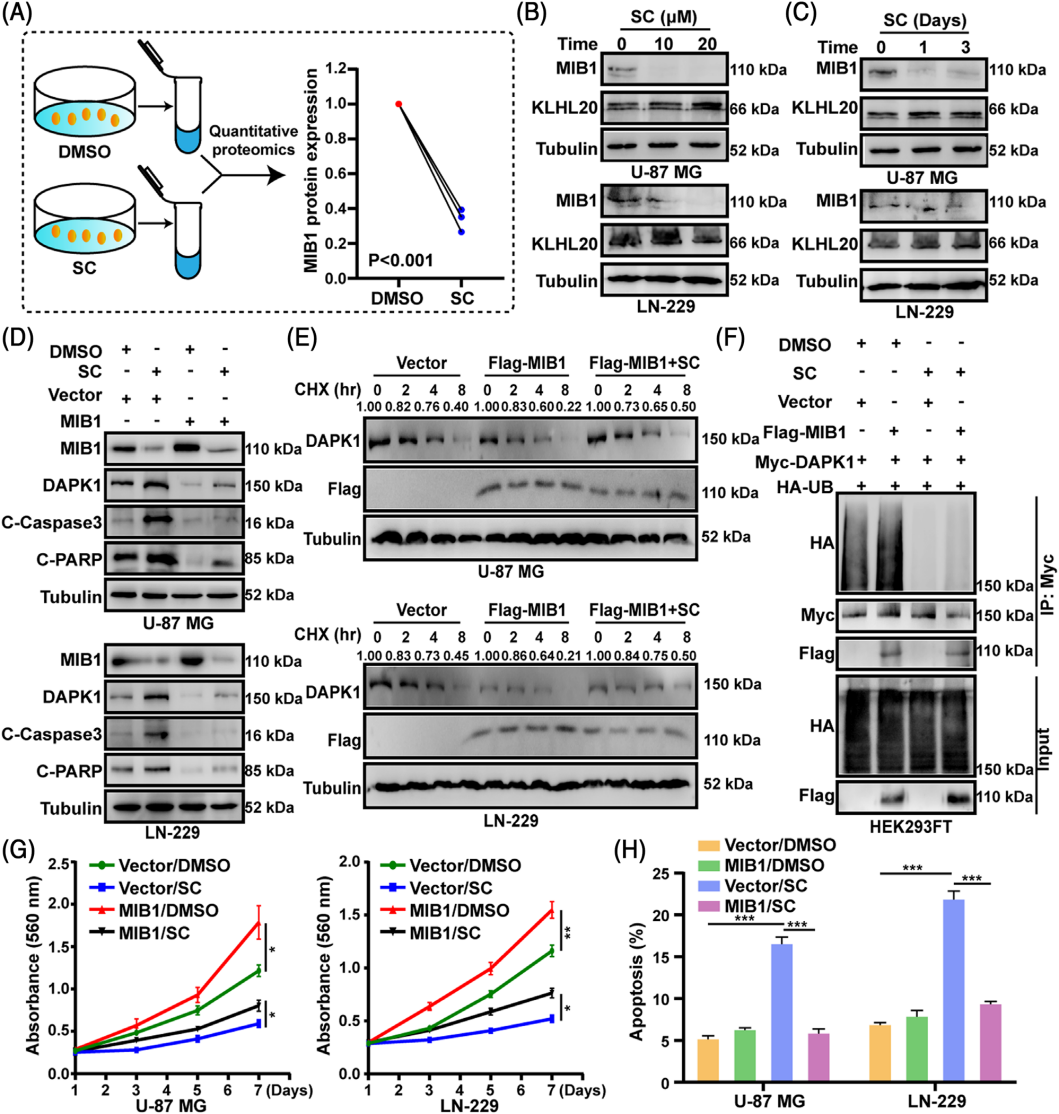
Sanggenon C (SC) increases the protein stability of DAPK1 through Mindbomb 1 (MIB1). (A) Quantitative proteomics in LN‐229 cells after treatment with SC (10 μM). DMSO was used as control. (B and C) Protein levels of MIB1 and KLHL20 were detected by Western blotting after U‐87 MG and LN‐229 cells were treated with the indicated concentrations and indicated times of SC. DMSO was used as control. (D) Western blot assays were performed to examine the protein expression level of MIB1, DAPK1, cleaved poly ADP‐ribose polymerase (C‐PARP), and cleaved caspase 3 (C‐Caspase3) in MIB1‐overexpression U‐87 MG and LN‐229 cells after treatment with SC (10 μM) or DMSO for 2 days. DMSO was used as control. (E) MIB1‐overexpression U‐87 MG and LN‐229 cells were treated with SC (10 μM) or DMSO and were then treated with cycloheximide (CHX) for the indicated times, and then were harvested and detect the DAPK1 turnover rate through Western blot analysis. The grayscale values were marked on the WB band, and 0 h was taken as the base for each group. DMSO was used as control. (F) The indicated plasmids were transfected into HEK293FT cells, and then treated with SC (10 μM) or DMSO. The ubiquitination of DAPK1 was detected through the Western blot experiments. DMSO was used as control. (G) MTT assays were performed to determine cell proliferation of MIB1‐overexpression U‐87 MG and LN‐229 cells treated with SC (10 μM) or DMSO for indicated times. DMSO was used as control. (H) MIB1‐overexpression U‐87 MG and LN‐229 cells were treated with SC (10 μM) or DMSO for 2 days and apoptosis was determined by flow cytometry. DMSO was used as control. The data were expressed as mean ± standard deviation (SD). Student's *t*‐test was performed to analyzed significance. ^*^ *p* < 0.05, ^**^ *p* < 0.01, ^***^ *p* < 0.001.

It has been reported that DAPK1 could be ubiquitinated and degraded by some E3 ubiquitin ligases, including MIB1 and KLHL20.^17^, ^18^ Analysis of the quantitative proteomics data revealed that the E3 ubiquitin ligase MIB1 was significantly down regulated after treatment with SC (Figure 5A and Table S4). Moreover, the protein expression levels of MBI1 and KLHL20 in GBM cell lines were detected, and the results showed that MIB1 was remarkably reduced after SC treatment, whereas no obvious change in the KLHL20 protein expression was observed (Figure 5B,C). Besides, we found a negative correlation between MIB1 and DAPK1 expression in GBM samples by immunohistochemistry analysis. (Figure S6A–C) To confirm the binding between MIB1 and DAPK1 in GBM cells, we performed the co‐immunoprecipitation analysis and found that MIB1 interacted with DAPK1 in GBM cells (Figure S7A). We further investigated the binding domains using truncation and/or deletion analysis, and the results showed that DAPK1 interacted with MIB1 through the death domain (Figure S7B). Moreover, we also performed the ubiquitination experiments using the ubiquitin wild‐type and mutant plasmids and found that MIB1 could not increase the ubiquitination of DAPK1 after mutation of K48, suggesting that MIB1 mediated the K48‐linked DAPK1 ubiquitination (Figure S8). We used MIB1 overexpressing GBM cells and either SC or DMSO treatment to further confirm whether SC increases the protein stability of DAPK1 by silencing MIB1. We discovered that the increase in DAPK1 expression brought on by SC could be somewhat inhibited by MIB1 overexpression in GBM cells (Figure 5D). Moreover, the MIB1‐overexpression groups experienced an increase in DAPK1 turnover, and treatment with SC partially reversed this effect (Figures 5E and S9). DAPK1 ubiquitination caused by MIB1 was reduced by SC, according to the ubiquitination analyses (Figure 5F). As a result, we surmised that the SC could be a downstream effector of the MIB1–DAPK1 axis in the cell proliferation and death of GBM cells. We treated MIB1 overexpressing GBM cells with SC or DMSO to test this theory. The results of the MTT and EDU tests showed that MIB1 overexpression greatly increased the proliferation of SC‐treated GBM cells (Figures 5G and S10A). TUNEL staining assays and flow cytometry analysis later proved that MIB1 overexpression in GBM cells inhibited the SC‐induced cell apoptosis (Figures 5H and S10B,C). Moreover, colony formation experiments were carried out to find out how SC affected the ability of GBM cells overexpressing MIB1 to form colonies. The results demonstrated that the colony formation capabilities of SC‐treated GBM cells were increased by overexpression of MIB1 (Figure S10D).

### 2.6 SC enhances the chemosensitivity to TMZ

**FIGURE 6:**
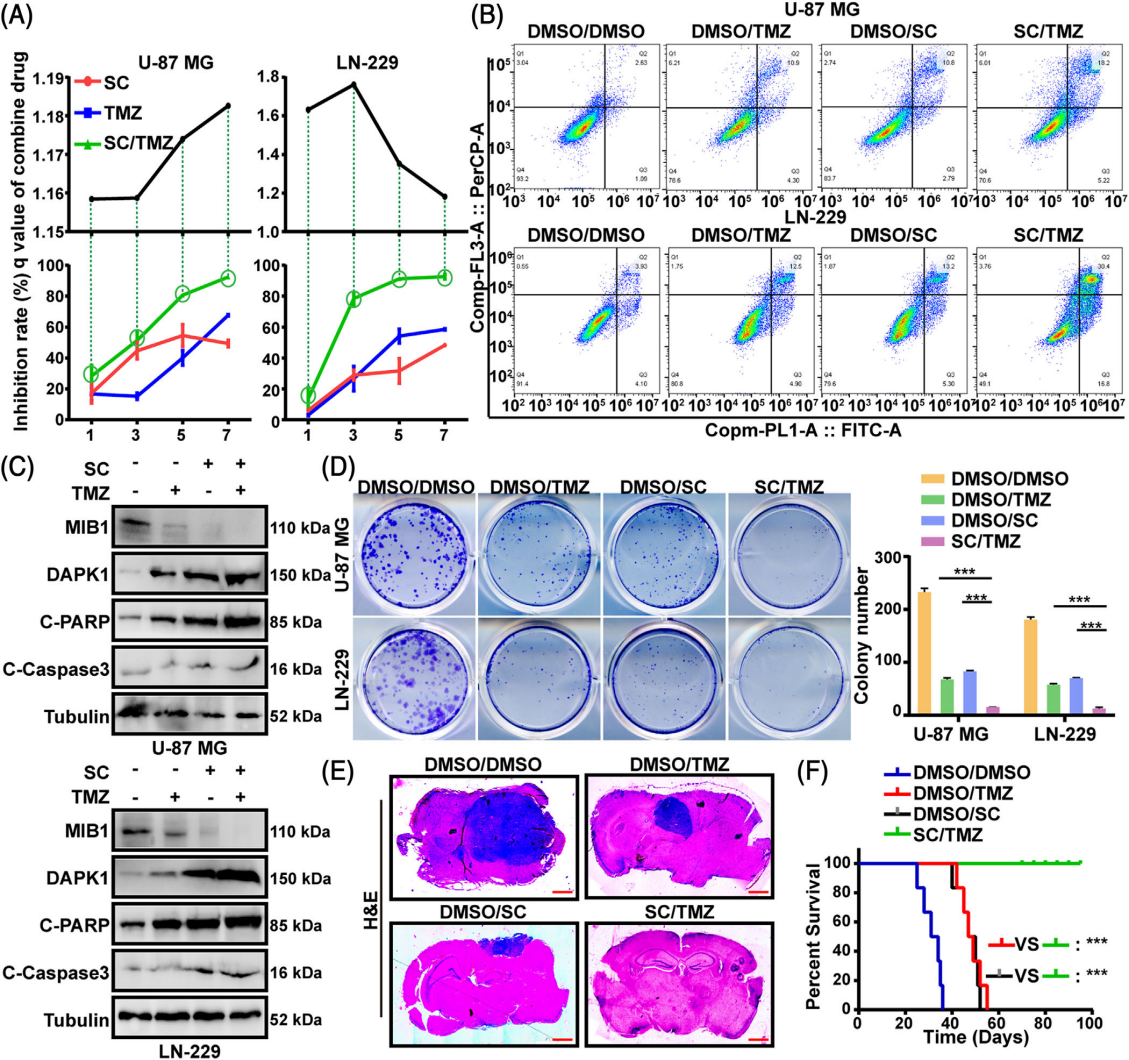
Sanggenon C (SC) enhances the chemosensitivity to temozolomide (TMZ). (A) Cell proliferation of U‐87 MG and LN‐229 cells treated with SC (10 μM), TMZ (300 μM), or SC (10 μM) + TMZ (300 μM) for indicated days were examined by MTT assays. DMSO was used as control. (B) U‐87MG and LN‐229 cells were treated with SC (10 μM), with or without TMZ (300 μm), and the apoptosis was detected with flow cytometry. DMSO was used as control. (C) U‐87MG and LN‐229 cells were treated with SC (10 μM), with or without TMZ (300 μM), and the protein expression level of Mindbomb 1 (MIB1), DAPK1, cleaved poly ADP‐ribose polymerase (C‐PARP), and cleaved caspase 3 (C‐Caspase3) were examined by Western blot assays. DMSO was used as control. (D) U‐87MG and LN‐229 cells were treated with SC (10 μM), with or without TMZ (300 μm), and the colony formation assays were used to determine the colony formation ability. DMSO was used as control. (E) Orthotopic implantation experiment was performed to assess the in vivo tumor growth capabilities of U‐87 MG cells. The mice were then treated with SC, with or without TMZ. Representative images of the hematoxylin and eosin (H&E) staining are presented. Scale bars = 2 mm. DMSO was used as control. (F) Overall survival of indicated mice and *p*‐value is indicated. The data were expressed as mean ± standard deviation (SD). Student's *t*‐test was performed to analyzed significance. ^*^ *p* < 0.05, ^**^ *p* < 0.01, ^***^ *p* < 0.001.

Temozolomide (TMZ), an imidazotetrazine prodrug used as a first‐line treatment for GBM, has shown limited clinical benefit primarily due to the poor response and resistance. There has not been any mention of TMZ and SC working together. By using Jin's formula and the *q*‐values ≥1.15, we saw in this study that the synergistic impact of TMZ and SC was observed throughout a broad range of times. The MTT assays showed that the TMZ and SC treatments improved the inhibition of cell proliferation (Figure 6A). Moreover, TMZ and SC together significantly increased the rate of cell apoptosis compared to the total of the two medicines used separately (Figures 6B and S11A). Western blot tests' findings, meantime, confirmed that in U‐87 MG and LN‐229 cells, the combined TMZ and SC treatment group exhibited increased DAPK1, C‐Caspase3, and C‐PARP protein expression and lower MIB1 protein expression than those treated with TMZ or SC alone (Figure 6C). Moreover, colony formation tests revealed that the combination of TMZ and SC could substantially lower the number of colonies of U‐87 MG and LN‐229 cells (Figure 6D). We performed the orthotopic implantation tests to further examine the therapeutic effects of the TMZ and SC combination in vivo. The findings showed that tumors found in mice treated with TMZ and SC together were considerably smaller than tumors found in mice treated with TMZ or SC alone (Figure 6D,E). In addition, the protein expression of MIB1 was clearly reduced by combination of TMZ and SC in the resultant tumors, whereas DAPK1 expression was increased after the combined treatment (Figure S11B). Furthermore, the treatment with TMZ + SC had a more blatant therapeutic effect on the mice with GBM when compared to treatment with TMZ or SC alone. Together, these results showed that SC alone did not have a substantial suppressive impact in vitro or in vivo compared to SC combination with TMZ.

## 3 DISCUSSION

Glioma is the most common brain cancer in adult humans, and GBM is the most devastating type due to molecular heterogeneity, infiltrating characteristics, the blood–brain barrier, and the highly immunosuppressive microenvironment.^19^ Nowadays, great advances have been made to help the neurosurgeons to define a comprehensive treatment including surgery, chemotherapy, radiotherapy, and immunotherapy. Unfortunately, GBM still has one of the worst 5‐year survival rates of all human tumors, and 90% of GBM patients have a recurrence within 6 months.^20^ Identification and development of novel therapeutic drugs could be a promising approach for tumor treatment.

The mulberry tree (mulberry) is a deciduous plant with excessive sensible value, and its leaves, branches, roots (bark), and fruits are broadly used in standard Chinese medicine. Its energetic components incorporate flavonoids, alkaloids, steroids, and coumarins, and these compounds have big anti‐oxidant, anti‐inflammatory, anti‐bacterial, anti‐tumor, and immunomodulatory effects.^9^, ^10^ SC is a herbal flavonoid that is extracted from mulberry white skin and has been said to have anti‐oxidative, anti‐inflammatory, and anti‐cancer outcomes.^21^ It has been suggested that SC can minimize the expression of proinflammatory genes (interleukin [IL]‐6, IL‐1β, and tumor necrosis factor‐α) and reactive oxygen species (ROS), while expanding the activities of anti‐oxidant enzymes in various stipulations.^14^ Recently, quite a few research suggested that SC has the attainability to suppress cell proliferation and set off apoptosis in tumors. For instance, SC suppressed gastric cancers cell increase and cancerogenesis, and resulted in cell apoptosis by blocking the activation of the extracellular‐signal‐regulated kinases (ERK) signaling pathway.^22^ Besides, remedy with SC drastically brought about cell arrest at G0/G1 section and promoted cell apoptosis in prostate cancers typically by way of the circHMGCS1‐miR‐205‐5p‐ErBB3 signaling pathway.^23^ A preceding document confirmed that SC promoted apoptosis of most colon cancer cells by suppressing the production of NO, the expression of iNOS, and the activation of ROS in the mitochondrial pathway.^24^ However, the impact of SC on GBM remains unclear.

This finding similarly consolidated the outstanding anti‐cancer exercise of SC in vivo and in vitro. According to the enrichment evaluation of transcriptomics and flow cytometry analysis, we discovered that remedy with SC remarkably precipitated cell cycle arrest at G0/G1 phase. Besides, apoptosis was once located in the SC‐treated GBM cells through the TUNEL staining and drift cytometry analysis. However, LC3B staining assays indicated that the SC remedy no longer set off autophagy in GBM cells. Herein, the molecular mechanisms underlying SC‐mediated cell proliferation and apoptosis of GBM cells have to be investigated. Our protein–protein quantitative proteomics and Western blot evaluation published that cure with SC notably decreased the expression of the E3 ubiquitin ligase MIB1, which acted as an E3 ubiquitin ligase to promote the proteasomal degradation of numerous proteins such as NRF2 and ST7 to feature in some cancers, thereby affecting tumor increase and improvement.^25^, ^26^ Besides, it has been mentioned that MIB1 ought to additionally act as an E3 ubiquitin ligase for DAPK1, a serine/threonine protein kinase concerned with numerous mobile development such as cell cycle and apoptosis.^17^ In this study, we determined that the growth of DAPK1 protein expression after SC therapy was once essential for inhibiting GBM cell proliferation and merchandising apoptosis. Western blot and ubiquitination assays confirmed that MIB1 responds to SC and has a poor correlation with DAPK1 protein stability. After MIB1 was once overexpressed, it may want to needless to say block the upregulation of DAPK1 precipitated with the aid of SC. Previous findings confirmed that SC was once in a position to accumulate proteasome substrate protein p27 in a dose‐dependent manner in general precipitated proteasome inhibition in murine H22 and P388 cell strains.^15^ Similarly, our results indicated that SC therapy decreased MIB1‐mediated DAPK protein degradation in a dose‐dependent manner in GBM cells. Herein, we speculated that SC commonly mediates the cell increase and apoptosis of GBM cells by regulating the MIB1–DAPK1 axis. The regulatory mechanism between SC and MIB1 requires a similar investigation.

Discovery of TMZ is a therapeutic drug for many tumor patients; however, the bad response and resistance limit the medical effect. The most interesting discovery of this study is that SC blended with TMZ exhibited a larger tumor‐inhibitory impact than TMZ on my own in U‐87 MG and LN‐229 cells. Therefore, SC can decorate the chemosensitivity of GBM cells to TMZ by regulating cell proliferation and apoptosis. These discoveries indicated that SC is a conceivable medical agent for cancer. In conclusion, we observed that SC superior the steadiness of DAPK1 protein by means of decreasing the expression of the E3 ubiquitin ligase MIB1 and fashioned a synergistic impact by using combining TMZ, thereby supplying new thoughts and new insights for the cure of GBM. However, there also have some limitations in this study, such as the mechanism that SC treatment increased TMZ sensitivity in GBM cells, which has not been well studied.

## 4 MATERIALS AND METHODS

### 4.1 Regents and antibodies

SC (Cat# DS0164) was obtained from LEIMEITIAN MEDICINE (Chengdu, China) and dissolved in DMSO. TMZ (Cat# HY‐17364) was purchased from MedChemExpress (NJ, USA). Anti‐CDK2 (Cat# 10122‐1‐AP), anti‐CDK4 (Cat# 11026‐1‐AP), anti‐CyclinE1 (Cat# 11554‐1‐AP), anti‐MIB1 (Cat# 11893‐1‐AP), anti‐Tubulin (Cat# 11224‐1‐AP), anti‐HA (Cat# 51064‐2‐AP), and anti‐DAPK1 (Cat# 25136‐1‐AP) antibodies were purchased from Proteintech (Wuhan, China). Anti‐cleaved PARP1 (Cat# 5625T), anti‐cleaved Caspase3 (Cat# 9664T), anti‐LC3B (Cat# 3868T) and anti‐Flag (Cat# 8146T) antibodies were purchased from Cell Signaling Technology (Boston, MA, USA). MTT (Cat# M5655), CHX (Cat# C7698), MG132 (Cat# M7449), and DMSO (Cat# D5879) were obtained from Sigma–Aldrich (St. Louis, MO, USA). 4′,6‐diamidino‐2‐phenylindole (DAPI) (Cat# C1002), the One Step TUNEL Apoptosis Assay Kit (Cat# C1089), the BeyoClick EdU Cell Proliferation Kit with Alexa Fluor 488 (Cat# C0071S), the Hematoxylin and Eosin (H&E) Staining Kit (Cat# C0105S), the bicinchoninic acid (BCA) Protein Assay Kit (P0012), the Cell lysis buffer for Western and immunoprecipitation (IP) (Cat# P0013), the Crystal Violet Staining Solution (Cat# C0121), horseradishperoxidase (HRP) goat anti‐mouse antibody (Cat# A0126), HRP goat anti‐rabbit antibody (Cat# A0208), and Alexa Fluor 488‐labeled Goat Anti‐Rabbit immunoglobulin G (Cat# A0423) were purchased from Beyotime (Shanghai, China). The transfection reagent Lipofectamine 2000 was obtained from Thermo Fisher Scientific (NY, USA).

### 4.2 Cell culture

GBM cell lines (LN‐229 and U‐87 MG), ordinary astroglia cells (SVGP12), and human embryonic kidney cells (HEK293FT) were purchased from the American Type Culture Collection (USA). The cell lines were examined to be mycoplasma‐free and cultured as previously described.^27^

### 4.3 Cell proliferation analysis

Using the MTT experiments, as previously described, cell viability was evaluated.^28^ Cells were sown onto 96‐well plates with three replicates and 1000 cells per well, and they adhered overnight. Then, 20 μL of MTT was applied to the cells at the appropriate times for a 2‐h incubation. Following the removal of the culture media and incubation with 150 μL of DMSO, the absorbance was determined using a microplate reader and a 560 nm wavelength.

### 4.4 EDU staining

EDU staining was used to identify cell proliferation in accordance with the manufacturer's recommendations. A total of 2 × 10^4^ cells were placed in 24‐well plates, cultivated for an overnight period, and then allowed to revert to their original state. Cells were first incubated for 2 h with 10 mM EDU, followed by 15 min with 4% paraformaldehyde (PFA), 10 min with 0.3% Triton X‐100, 1 h with 5% bovine serum albumin (BSA), and 30 min with Click reaction cocktails. Before being examined under a microscope, the nuclei were dyed with DAPI for 30 min at room temperature.

### 4.5 Flow cytometry

Cells were digested, centrifuged, and then resuspended in phosphate‐buffered saline (PBS) buffer after being treated for 48 h with SC or DMSO. Cells were fixed in 75% ethanol for at least 24 h prior to cell cycle examination, which was followed by PI and RNase labeling. Then, using flow cytometry, cells were found. Each group included three copies. Cells were labeled with FITC‐Annexin V and PI for 30 min, and flow cytometry was used to identify the cells. Annexin V‐FITC/PI Apoptosis Detection Kit was used after cell apoptosis labeling (Beyotime).

### 4.6 TUNEL staining

On coverslips, 2 × 10^4^ cells were cultured before being stained using the One Step TUNEL Apoptosis Assay Kit (Beyotime). Cells were first treated for 48 h with SC or DMSO, and then incubated with 4% PFA for 30 min, 0.3% Triton X‐100 for 5 min, 5% BSA for 1 h, and the TUNEL test solution (50 L/well) for 60 min in the dark. Lastly, an anti‐fluorescence quenching liquid was used to seal the coverslips, and a fluorescence microscope was used to view the results. The emission wavelength (red fluorescence) was 570 nm.

### 4.7 Transfection and infection

Short hairpin (sh) RNA‐based silencing of DAPK1 was recombined into pLKO.1‐puro plasmid, and the sequences are listed in Table S1. Flag‐tagged MIB1 was purchased from Yubio (Wuhan, China). For transfection, the indicated plasmids were transfected into HEK293FT cells using Lipofectamine 2000 according to the manufacturer's instructions. Viral supernatant or cells were harvested 2 days later. For infection, GBM cells were infected by treatment with viral supernatant and polybrene (Sigma, USA). After two rounds of infection, cells were stably selected and pooled with puromycin (Sigma).

### 4.8 Western blot

Cells were extracted using a cell scraper, washed three times in PBS buffer, and then lysed on ice in RIPA cell lysis buffer. Using the BCA protein assay kit and 60 μg of protein combined with five loading buffer that was cooked in 100°C water baths for 15 min, protein concentration was calculated. After that, trans blotting and SDS‐PAGE were performed on the samples. After blocking the PVDF membrane (Millipore, Germany) with 5% BSA for 2 h at room temperature, primary and secondary antibodies that were HRP‐linked were incubated. Once the membrane was exposed, an ECL detection system did the rest (Clinx, Shanghai).

### 4.9 Label‐free analysis

After treatment with SC or DMSO for 2 days, LN‐229 cells were harvested, and detected by label‐free quantitative proteomic analysis (Shanghai Applied Protein Technology Biotechnology Corporation), through protein extraction, peptide enzymatic hydrolysis, liquid chromatography–tandem mass spectrometry data collection, database retrieval, etc. In the screening of significantly differentially proteins, the number of upregulated and downregulated proteins between groups was compared using fold change >2.0‐fold (upregulated >2.0‐fold or downregulated <0.5‐fold) and *p*‐value <0.05 as the standard.

### 4.10 Ubiquitination assay and protein turnover assay

The specified plasmids were co‐transfected into the HEK293FT cells for the ubiquitination test. According to a prior report, cells were collected and subjected to co‐immunoprecipitation (Co‐IP) and Western blot analysis after being incubated with MG132 for 8 h.^19^

The relevant GBM cells were treated with SC or DMSO for the protein turnover test. Cells were extracted and subjected to Western blot analysis following treatment incubation with 100 g/mL CHX for the specified durations.

### 4.11 Quantitative PCR

Cells were lysed with TRIzol reagent, chloroform was added, and mixed gently to remove the organic phase. The RNA was precipitated by adding isopropanol to the aqueous phase. The RNA precipitate was washed with 75% ethanol and dissolved in TE buffer. RNA concentration was determined and reverse transcription was performed using 2 μg RNA. The reverse transcription was performed using GoScript (Promega, USA) reverse transcriptional system. SYBR qPCR SuperMix Plus was used to perform qRT‐PCR. The internal reference was GAPDH. Table S2 lists the primers used in this study.

### 4.12 Immunofluorescence

The 24‐well plates (2 × 10^4^ cells/well) with a round coverslip in place were then filled with GBM cells. Cells were cultured for 24 h before being fixed with 4% PFA for 15 min and treated with 0.3% Triton to increase cell permeability. Cells were first blocked with 10% BSA before being treated with an anti‐LC3B antibody and goat anti‐rabbit secondary antibody that had been Alexa Fluor 488‐labeled. DAPI was used to label the nuclei before microscopy.

### 4.13 Colony formation assay

With the use of a colony formation experiment, the impact of SC on the capacity of GBM cells to form colonies was investigated. In a six‐well plate, 1000 cells and SC were put into each well. The colonies were dyed with crystal violet after 2–3 weeks of culture, and then the colonies were counted by ImageJ software in each well.

### 4.14 H&E staining

The tissue samples were sequentially fixed, dehydrated, embedded, and sectioned. Then, the sections have been stained with the H&E Staining Kit (Beyotime) in accordance with the manufacturer's instructions.

### 4.15 Animal studies and animal ethics

The experiments were performed as previously described.^27^ Briefly, 36 NOD/SCID mice (4‐week‐old female, Slike Jingda Laboratory Animal Co., Ltd., Hunan, China; animal qualification number: SCXK‐2019‐0004) were housed in the SPF room. U‐87 MG cells (1 × 10^5^ cells) were intracranially injected slowly into the brains of each mouse. After 10 days, mice were injected intraperitoneally with SC or TMZ (30 mg/kg/day) every 2 days for 20 days. DMSO injections were administered to control mice. Before collecting brains, mice have been anesthetized with isoflurane to relieve pain. Randomized and single‐blind strategies have been used for measurement, and the tumor quantity evaluation was once as described before.^27^ The animal experiments in this study were approved by the Institutional Animal Care and Use Committee of the Southwest University (ethics approval serial number: IACUC‐20230320‐04), and carried out in conformity with the Guide for the Care and Use of Laboratory Animals (Ministry of Science and Technology of China, 2006).

### 4.16 Jin's formula

We assessed the drug combined effect in vitro through Jin's formula. The Jin's formula is as follows:

$$q=\frac{E(A+B)}{\left(\mathrm{EA}+\mathrm{EB}-\mathrm{EA}\times \mathrm{EB}\right)}$$

where *q* is the efficiency index, *E*(*A* + *B*) is the combined inhibition rate, EA is the drug A inhibition rate, and EB is the drug B inhibition rate (*q* < 0.85: antagonism effect, 0.85 ≤ *q* < 1.15: superosition effect, *q* ≥ 1.15: synergistic effect)

### 4.17 Statistics analysis

All data were presented three times independently, and GraphPad Prism 8 was used to process the data, which are presented as mean ± standard deviation. Significant difference analysis was performed on independent samples using the Student's unpaired *t*‐test, and the results were considered significant as *p* < 0.05 (^*^ *p* < 0.05, ^**^ *p* < 0.005, ^***^ *p* < 0.0005).

## AUTHOR CONTRIBUTIONS

H.C., J.H., Y.S., M.X., X.W., Y.D., J.S., Z.L., and H.C. have participated in investigation, methodology, and validation of data presented in this article. H.C. and J.H. are responsible for formal analysis of data and writing and editing of this manuscript. Z.L. and H.C. read and revised this manuscript. All authors have read and approved the final manuscript.

## CONFLICT OF INTEREST STATEMENT

The authors declare they have no conflicts of interest.

## ETHICS STATEMENT

The animal study protocol was approved by Institutional Animal Care and Use Committee of Southwest University for studies involving animals. Ethics approval serial number: IACUC‐20230320‐04.

## Supporting information

Supplementary Information

## Data Availability

The datasets used and/or analyzed during the current study are available from the corresponding author upon reasonable request.
