# Supplementary material for: Sanggenon C inhibits cell proliferation and induces apoptosis by regulating the MIB1/DAPK1 axis in glioblastoma
Source: MedComm (2020). 2023 Jun 19;4(4):e281. doi: 10.1002/mco2.281 (PMC10279945; doi:10.1002/mco2.281)
Supplement: Supplementary file 1 — Supplementary Information [file MCO2-4-e281-s001.pdf]

Supplementary Table 1. The shRNA sequences were listed as below:

shDAPK1-1-F	CCGGCCACGTCGATACCTTGAAATTCTCGAGAATTTCAAGGTATCGACGTGGTTTTTG
shDAPK1-1-R	AATTCAAAAACCACGTCGATACCTTGAAATTCTCGAGAATTTCAAGGTATCGACGTGG
shDAPK1-2-F	CCGGCGACATCCAGAACGCTTATTTCTCGAGAAATAAGCGTTCTGGATGTCGTTTTTG
shDAPK1-2-R	AATTCAAAAACGACATCCAGAACGCTTATTTCTCGAGAAATAAGCGTTCTGGATGTCG
shDAPK1-3-F	CCGGCCTTGCTTCTTACTGATAATTCTCGAGAATTATCAGTAAGAAGCAAGGTTTTTG
shDAPK1-3-R	AATTCAAAAACCTTGCTTCTTACTGATAATTCTCGAGAATTATCAGTAAGAAGCAAGG

Supplementary Table 2. Primer pairs for real-time PCR:

CDK2-F	CCAGGAGTTACTTCTATGCCTGA
CDK2-R	TTCATCCAGGGGAGGTACAAC
CDK4-F	ATGGCTACCTCTCGATATGAGC
CDK4-R	CATTGGGGACTCTCACACTCT
CyclinE1-F	GCCAGCCTTGGGACAATAATG
CyclinE1-R	CTTGCACGTTGAGTTTGGGT
DAPK1-F	GAGTTTGTCGCTCCTGAGATAGT
DAPK1-R	GCTTAGTGTCTCCAAGAAATGGG
GAPDH-F	CTGGGCTACACTGAGCACC
GAPDH-R	AAGTGGTCGTTGAGGGCAATG

Supplementary Table 3. The most regulated gene in transcriptomics (Top 10):

Upregulated		Downregulated	
Gene name	Fold change (SC/DMSO)	Gene name	Fold change (SC/DMSO)
KLHDC7B	2180	IGFBP5	0.0174893
GPR1	541	FAM111B	0.0130112
BHLHA15	166.25	ID1	0.0422597
INHBE	129.16667	FABP7	0.0402906
STC2	41.587234	E2F2	0.029654
LURAP1L	51.870968	AATK	0.0357583
BEX2	53.666667	KCNJ10	0.0294118
AKNA	38.84	ID3	0.057945
ADM2	42.121212	RRM2	0.0656356
SCN9A	104.33333	E2F8	0.0269461

Supplementary Table 4. The most downregulated gene in proteomic (Top 10):

Upregulated		Downregulated	
Gene name	Fold change (SC/DMSO)	Gene name	Fold change (SC/DMSO)
PLEKHA2	390.06596	MIB1	0.3049787
LYST	167.20783	DAXX	0.2871708
ATF7IP	100.55276	GRPEL2	0.2843688
GTPBP2	85.13368	TTC19	0.2839056
RAB43	72.209731	PCCB	0.2808041
EEF1AKMT2	67.651357	PEX13	0.2799301
C1orf174	44.442272	CCS	0.2790116
FASTKD5	44.302443	IL13RA2	0.2737214
OXLD1	41.734758	SMC6	0.2723008
H2AZ2	38.340521	TGOLN2	0.2715005

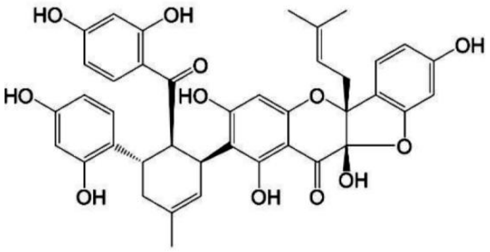

Sanggenon C (SC)

Supplementary Figure1. The chemical structure of SC.

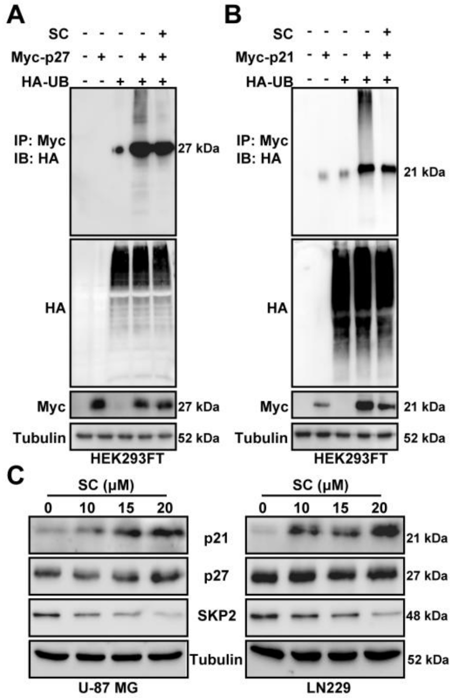

Supplementary Figure2. (A) WB analysis was to detect the effect of SC on P27 ubiquitination. (B) WB analysis was used to detect the effect of SC on P21 ubiquitination. (C) The expression of p27, p21 and SKP2 in LN-229 and U-87 MG cells treated with SC concentration gradient was detected by WB.

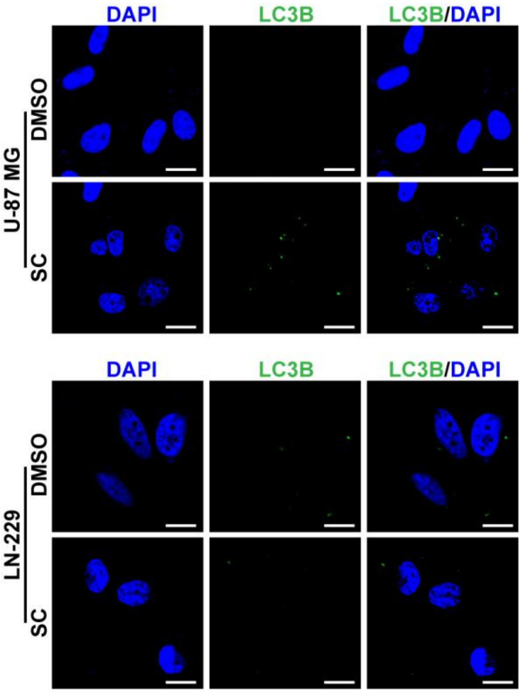

Supplementary Figure3. A immunofluorescence staining of LC3B (green) in U-87 MG and LN-229 cells treated with or without SC (10μM) for 2 days. The nuclei were counter stained with DAPI (blue). Scale bars=10μm.

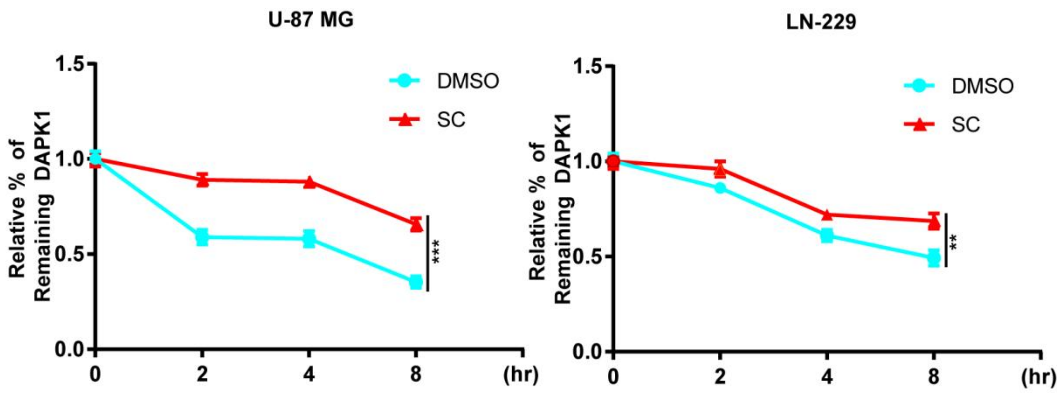

Supplementary Figure4. U-87 MG and LN-229 cells were treated with SC (10μM) or DMSO and were then treated with CHX (100μg/mL) for the indicated times, and then were harvested and detect the DAPK1 turnover rate through western blot analysis. Western blot gray value is analyzed and quantified, and 0h was taken as the base for each group. DMSO was used as control.

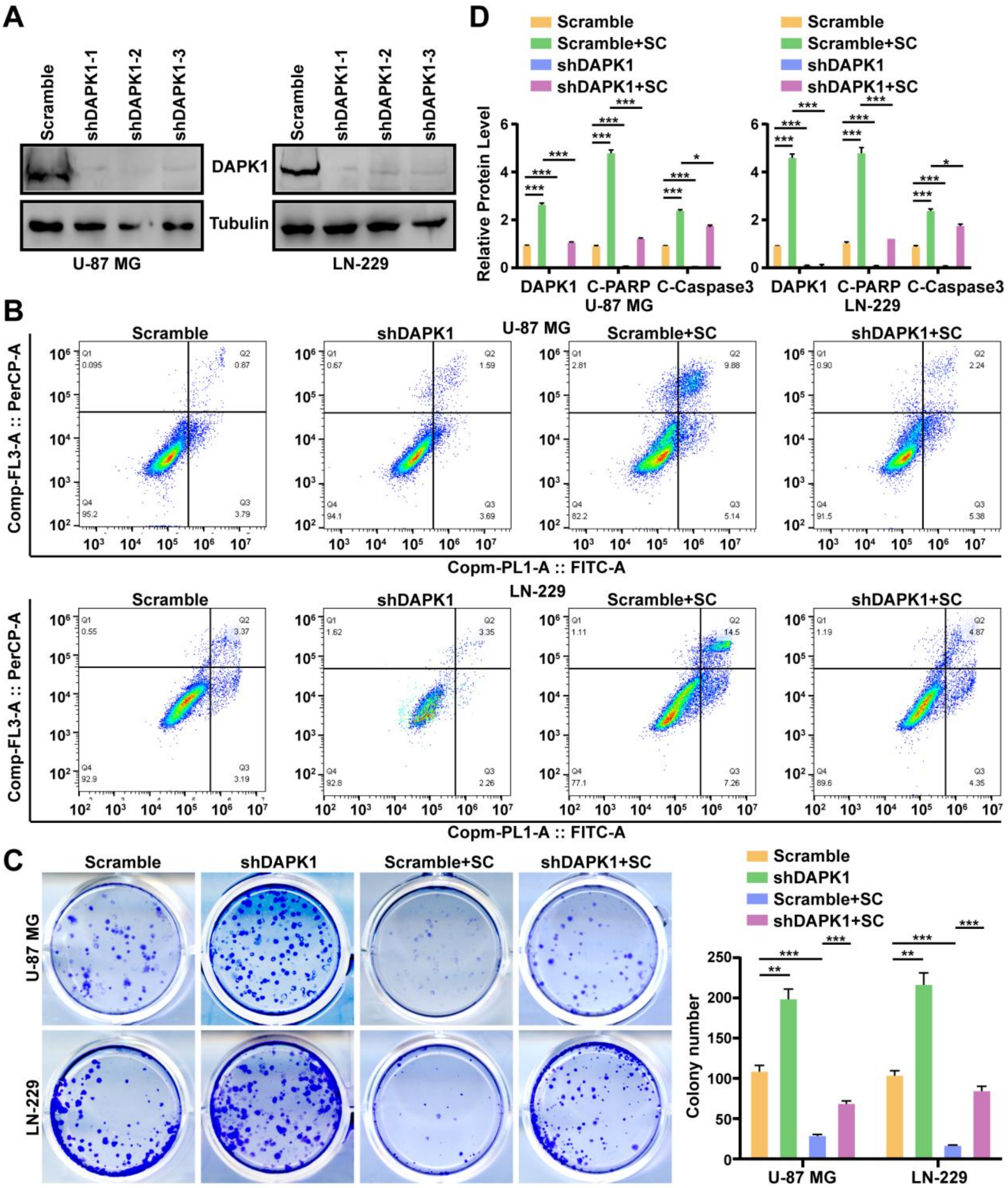

Supplementary Figure5. (A) Western blot analysis were used to detect the protein expression level of DAPK1 in DAPK1-knockdown and control cells. (B) shDAPK1-downregulation U-87 MG and LN-229 cells were treated with SC (10μM) or DMSO for 2 days and apoptosis was determined by flow cytometry. (C) The colony formation experiments were performed to assess the colony formation ability of shDAPK1-downregulation U-87 MG and LN-229 cells treated with SC (10μM) or DMSO. (D) Indicated protein levels are estimated based on the grayscale value of protein bands and normalized with the grayscale value of Tubulin bands.

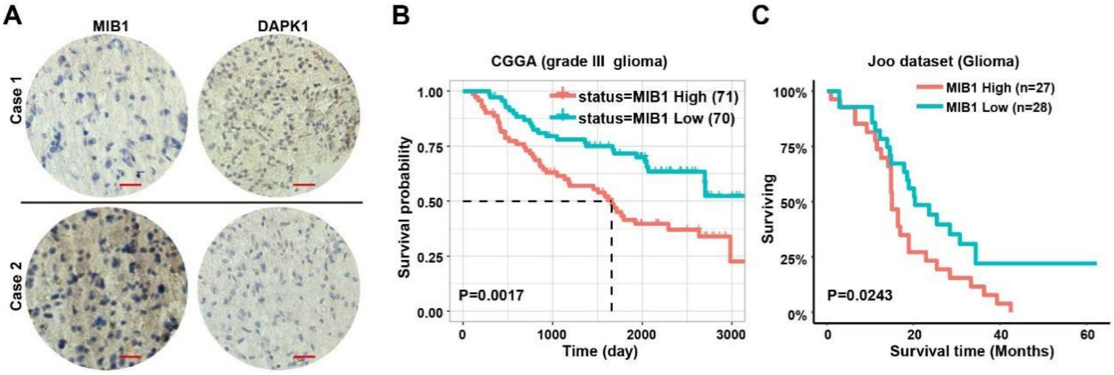

Supplementary Figure6. (A) MIB1 and DAPK1 expression in Glioblastoma multiforme clinical samples. (B) The relationship between MIB1 expression and prognosis of glioma patients was analyzed by CGGA database. (C)The relationship between MIB1 expression and prognosis of glioma patients was analyzed by Joo dataset.

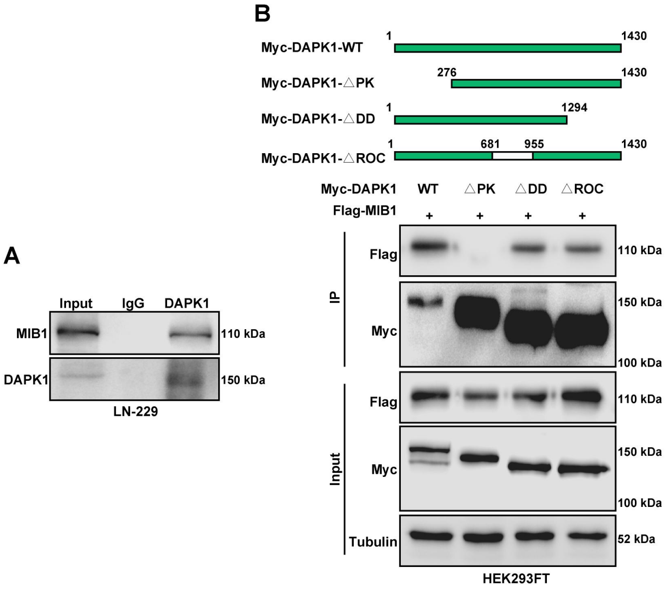

Supplementary Fig 7. (A) Immunoprecipitation assay to detect the interaction between DAPK1 and MIB1. (B) The domain of DAPK1 and the interaction between MIB1.

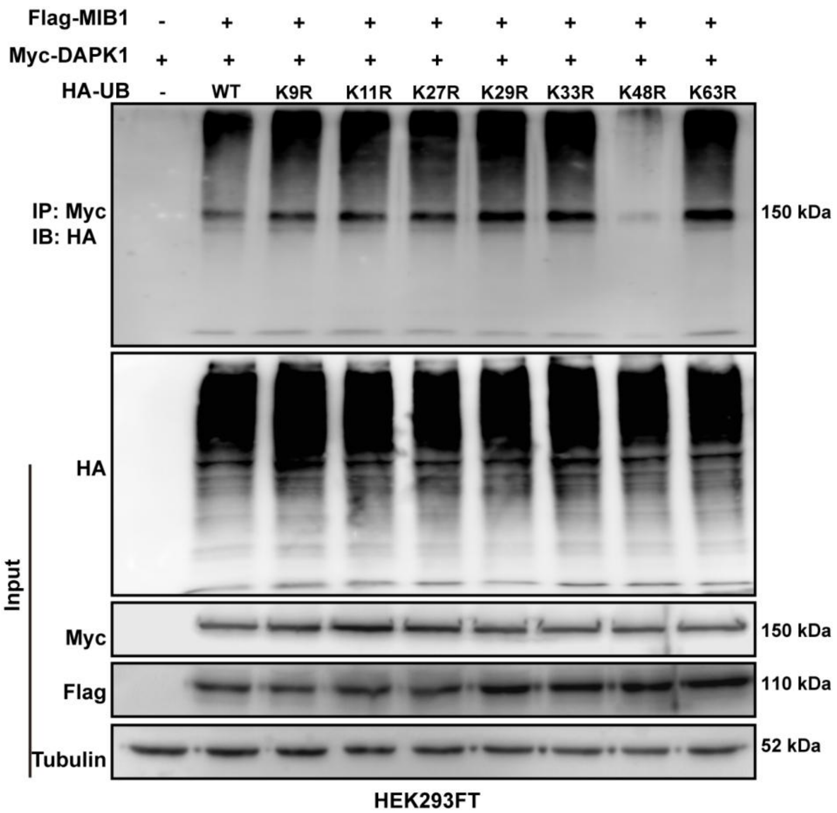

Supplementary Fig 8. Detection of MIB1 ubiquitination sites in DAPK1 by Western blot assay and ubiquitination assay.

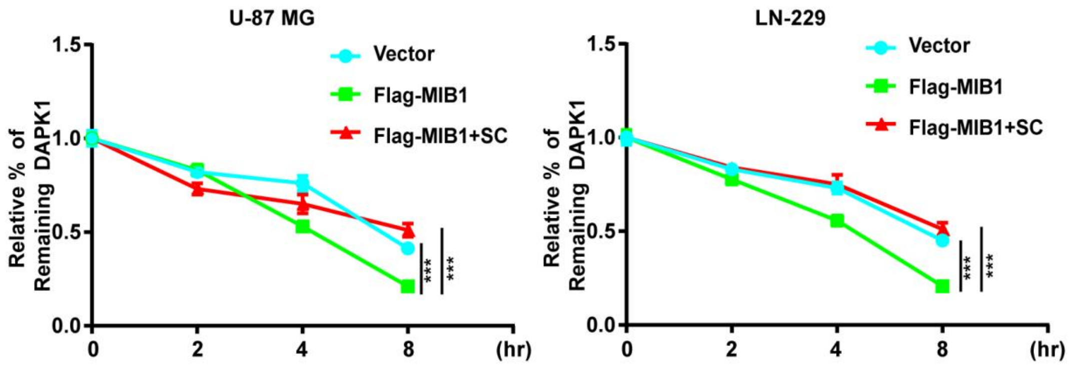

Supplementary Fig 9. MIB1-overexpression U-87 MG and LN-229 cells were treated with SC (10μM) or DMSO and were then treated with CHX for the indicated times, and then were harvested and detect the DAPK1 turnover rate through western blot analysis.Western blot gray value is analyzed and quantified, and 0h was taken as the base for each group. DMSO was used as control.

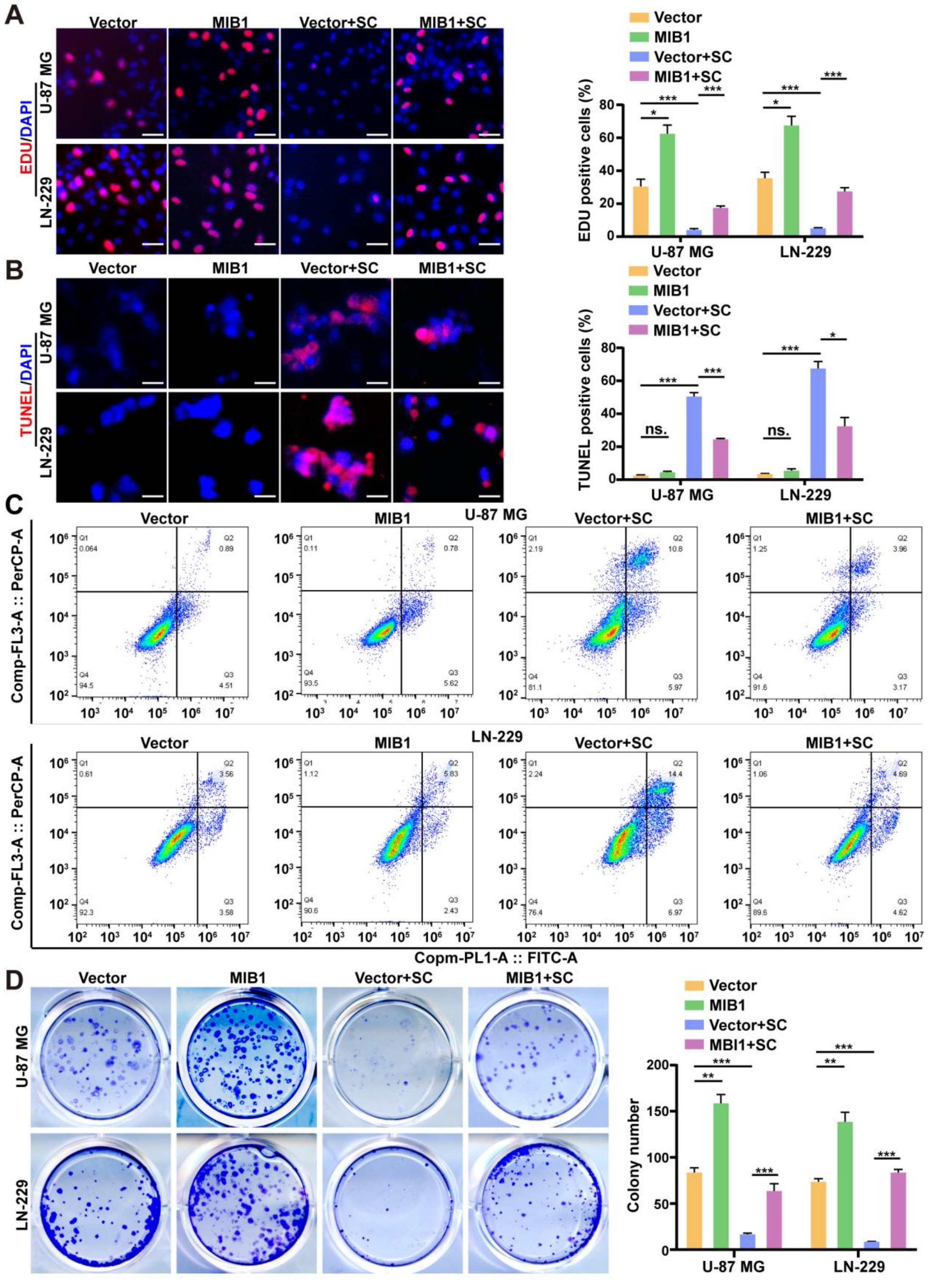

Supplementary Figure 10. (A) EDU-positive cells in MIB1-overexpression U-87 MG and LN-229 cells after treatment with SC (10μM) or DMSO.Scale bars=100μm. (B) TUNEL-positive cells in MIB1-overexpression U-87 MG and LN-229 cells after treatment with SC (10μM) or DMSO. Scale bars=50μm. (C) MIB1-overexpression U-87 MG and LN-229 cells were treated with SC (10μM) or DMSO for 2 days and apoptosis was determined by flow cytometry. (D) The colony formation experiments were performed to assess the colony formation ability of MIB1-overexpression U-87 MG and LN-229 cells treated with SC (10μM) or DMSO.

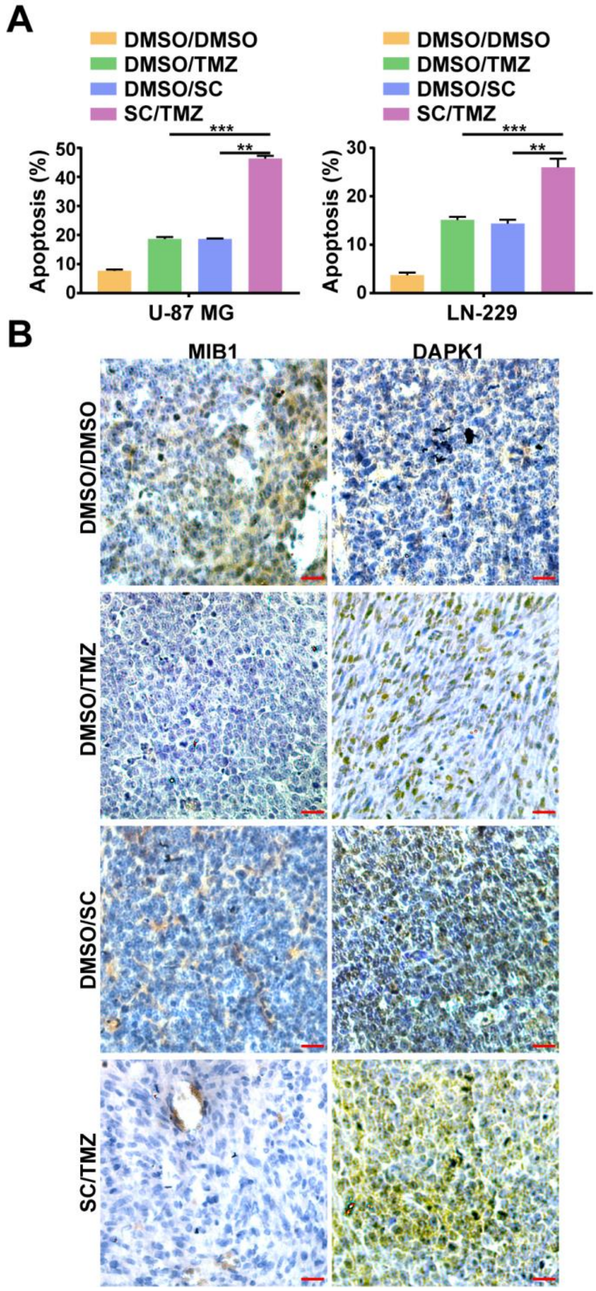

Supplementary Fig 11. (A)U-87MG and LN-229 cells were treated with SC (10μM), with or without TMZ (300μm), and the apoptosis was detected with flow cytometry. DMSO was used as control.(B) Expression of MIB1 and DAPK1 in Glioblastoma multiforme, and detection of synergistic effects of SC and TMZ.
